# Inferring Models of Bacterial Dynamics toward Point Sources

**DOI:** 10.1371/journal.pone.0140428

**Published:** 2015-10-14

**Authors:** Hossein Jashnsaz, Tyler Nguyen, Horia I. Petrache, Steve Pressé

**Affiliations:** 1 Physics Dept., Indiana Univ. - Purdue Univ. Indianapolis, Indianapolis, IN, 46202, United States of America; 2 Stark Neuroscience Institute, Indiana Univ. School of Medicine, Indianapolis, IN 46202, United States of America; 3 Dept. of Cell and Integrative Physiology, Indiana Univ. School of Medicine, Indianapolis, IN 46202, United States of America; A*STAR Bioinformatics Institute, SINGAPORE

## Abstract

Experiments have shown that bacteria can be sensitive to small variations in chemoattractant (CA) concentrations. Motivated by these findings, our focus here is on a regime rarely studied in experiments: bacteria tracking point CA sources (such as food patches or even prey). In tracking point sources, the CA detected by bacteria may show very large spatiotemporal fluctuations which vary with distance from the source. We present a general statistical model to describe how bacteria locate point sources of food on the basis of stochastic event detection, rather than CA gradient information. We show how all model parameters can be directly inferred from single cell tracking data even in the limit of high detection noise. Once parameterized, our model recapitulates bacterial behavior around point sources such as the “volcano effect”. In addition, while the search by bacteria for point sources such as prey may appear random, our model identifies key statistical signatures of a targeted search for a point source given any arbitrary source configuration.

## Introduction

Bacteria sense chemoattractants (CA) or chemorepellents (CR) through a sequence of stochastic detection events at their chemoreceptors [1, 2] and convert temporal variations in the number of detection events into a directional bias [3–5].

Experiments report a sensitivity in *E. coli*’s response to CAs down to a few detection events [6, 7]. For instance, bacterial runs in *E. coli* can be substantially lengthened (by 30%) even in *nM* gradients [6, 7].

This suggests that the external noise in the stochastic detection process—the ‘hit’ events at the bacterium’s chemoreceptors—may affect a bacterium’s search strategy for food.

Here, we are motivated by this work to tackle a regime rarely studied in the literature [8]: how bacteria detect and move toward point food sources—such as patches of CAs [9] or even prey or lysed cells [10]—where the fluctuations in the number of hits (i.e. external noise) may be very high especially far from the source. Beyond high fluctuations in CA concentration away from the source, the mean CA concentration emitted from the point source varies very rapidly near the source. What is more, point sources—which generate non-monotonic CA/CR concentration profiles—can be dynamical (if sources are moving bacterial prey) and be present in large numbers. These defining characteristics of the CA profile [high fluctuations away from the source, rapidly varying mean near the source]—different from the well-defined CA/CR gradient [2, 4, 11, 12]—give rise to unique bacterial dynamical behavior near the point source.

Our goal is to build a ‘top-down’ model valid across bacterial species that will describe how bacteria respond to stochastic detection events (hits) to locate point sources. One of the main goals of our model will be to identify—from the dynamics of bacteria near the unique profile setup by point sources—statistical signatures of targeted search by bacteria toward (or away) from point sources. This will help distinguish a random search strategy—as, for example, is believed to be the case for the hunting strategy of the model bacterial predator *Bdellovibrio bacteriovorus*—from an otherwise targeted search for prey [13].

By contrast to the point source regime, much of what is known about chemotaxis is derived from studies on *E. coli* [3, 5, 7, 11, 14–21] and often in well controlled, *μM*, CA gradients [2, 4, 22, 23]. For instance, it is known that *E. coli* shows an approximate two-state *run-and-tumble* dynamics [4, 11, 14, 23, 24] generated by the intermittent coalescence and unbundling of its flagella which, in turn, is induced by the rotational bias of motors located at each flagellum’s base [4, 11, 14, 15]. This simplified model may be nuanced by the stochastic reality that not all motors rotate in lockstep [15].

As opposed to other modeling approaches [25], our model will not assume a two-state (*run-and-tumble*) dynamics from the onset. Rather, our model will be constructed starting from simple general principles: *i)* adaptation (which is the sensitivity to relative rather than absolute changes in CA/CR known to hold in *E. coli* [2, 22, 24]) and *ii)* stochastic signal integration over time through a memory (alternatively ‘response’) function entirely determinable from the data [16, 26].

One key strength of our approach will be to show that—even in the limit of large noise—all model parameters can be directly inferred from single cell tracking data using a maximum likelihood approach.

Once parametrized using one food source configuration (even if it is an artificially well-controlled source), we will show that the parametrized model is transferable to different and even poorly controlled food source configurations and can be used to make predictions about dynamical behavior near any source configuration.

## Materials and Methods

### The Model

#### Modeling a point source

We consider a point food source, located at **r**_*s*_, from which particles are emitted with rate R. The particles diffuse away from the source according to the following normal diffusion equation [27]

$$\frac{\partial c(r_{j}|r_{s};t)}{\partial t}=D△c\left(r_{j}|r_{s};t\right)-\frac{1}{\tau }c\left(r_{j}|r_{s};t\right)+R\delta \left(r_{j}-r_{s}\right)$$ (1)

where *τ* is the particle decay time constant (which, on physical grounds, can be very large), *D* is the particle diffusion coefficient and △ is the Laplacian. In the most general case, the location of the source is a function of time, **r**_*s*_ = **r**_*s*_(*t*). The detection rate (called hit rate), *R*(**r**_*j*_ | **r**_*s*_;*t*), by the searcher of those particles (the bacterium) is obtained from *c*(*r*_*j*_∣*r*_*s*_;*t*) [27].

Here we illustrate the explicit form for *R*(**r**_*j*_ | **r**_*s*_;*t*) for a stationary concentration profile with open boundary conditions [27]. In 3 dimensions, we have

$$R\left(r_{j}|r_{s}\right)=4\pi aDc\left(r_{j}|r_{s}\right)=\frac{aR}{|r_{j}-r_{s}|}\text{exp}(-\frac{|r_{j}-r_{s}|}{\lambda })$$ (2)

where **r**_*j*_ is the location of the searcher, *a* is the searcher’s radius and $\lambda =\sqrt{D\tau }$.

In general, the number of hits, *h*_*j*_, detected by the searcher at position **r**_*j*_ over some time interval [*t*, *t* + Δ*T*] is Poisson distributed

$$P(h_{j})=\frac{{\left(\int_{t}^{t+\Delta T}dt^{\prime }R(r_{j}|r_{s};t^{\prime })\right)}^{h_{j}}}{h_{j}!}\text{exp}\left(-\int_{t}^{t+\Delta T}dt^{\prime }R(r_{j}|r_{s};t^{\prime })\right).$$ (3)

For a fixed source, the above simplifies to

$$P(h_{j})=\frac{{\left(△TR(r_{j}|r_{s})\right)}^{h_{j}}}{h_{j}!}\text{exp}\left(-△TR(r_{j}|r_{s})\right).$$ (4)

#### Modeling the bacterium

Bacteria do not measure gradients directly. Rather, they detect stochastic hits at their chemoreceptors and use this hit information to bias their random walk [24, 28].

For this reason, we define a transition probability, *p*(**r**_*j* + 1_|{**r**_*i*_, *h*_*i*_}_*i*≤*j*_), for a bacterium to move to a new position **r**_*j*+1_ which occurs at every time step. This transition probability is conditioned on the bacterium’s previous hit history (which is supplied by the conjugate pairs of variables {**r**_*i*_, *h*_*i*_}_*i*≤*j*_).

Our transition probability, *p*(**r**_*j* + 1_|{**r**_*i*_, *h*_*i*_}_*i*≤*j*_), is a general mathematical object that is not specific to any bacterial species. To help make the form for *p*(**r**_*j* + 1_|{**r**_*i*_, *h*_*i*_}_*i*≤*j*_) concrete, we draw from the following physical observations:

1. Bacteria show adaptation [2, 19, 29, 30] (that is, they respond to relative changes in hits not absolute changes) and compare hits at different locations to bias their search [4]. Thus, their new position, **r**_*j*+1_, depends on ▽ log *h* not simply log *h* or *h*. We define ▽ log *h* for a discrete *h* further below.
2. Bacteria are subject to random, Brownian, motion [31, 32] as well as internal noise originating from the stochasticity in relaying their chemotactic signal (such as, for example in *E.coli*, binding of active CheY-P to the flagellar motor complex which, in turn, biases the motor’s rotational direction) [6, 22]. Therefore bacteria can—at best—select their new position and direction from their current position and their past history to within some precision we call *σ*.
3. Bacteria incorporate previous hit information using a memory function, described below, labeled {*α*} which we will extract from the data [16, 26].

These considerations motivate the following general form for the transition probability

$$p(r_{j+1}|{\left\{r_{i},h_{i}\right\}}_{i\leq j})=N\text{exp}\left(-\frac{{\left(r_{j+1}-r_{j}-\sum_{i=0}^{m}\alpha_{i}f_{j-i}\right)}^{2}}{2\sigma^{2}}\right),$$ (5)

where the coefficients {*α*}—having dimensions of length—determine precisely how previous hit information biases the cell’s most likely future position **r**_*j* + 1_ and

$$f_{j}\equiv \frac{\left(r_{j}-r_{j-1}\right)}{|r_{j}-r_{j-1}|}\cdot \frac{\left(h_{j}-h_{j-1}\right)}{h_{j}}\equiv \nabla \log h_{j}$$ (6)

where, as before, *h*_*j*_ are the number of hits at position **r**_*j*_ where, to be clear, the hits are the number of stochastic detections of CA/CR molecules by outer membrane chemoreceptors. For convenience, we can write *p*(**r**_*j*+1_|{**r**_*i*_, *h*_*i*_}_*i*≤*j*_) as *p*(**r**_*j* + 1_|{**r**_*i*_, Δ log *h*_*i*_}_*i*≤*j*_). The normalization constant is $N=\int dr_{j+1}p\left(r_{j+1}|{\left\{r_{i},\nabla \log h_{i}\right\}}_{i\leq j}\right)$ and, finally, *m* (the ‘memory’) determines how far into the past hit information is considered by the bacterium in selecting its future position.

The vector

$$\frac{r_{j}-r_{j-1}}{|r_{j}-r_{j-1}|}$$ (7)

introduced in Eq (6) determines the direction in which the motion is being biased.

As a technical aside, we note that if *h*_*j*_ is very small—and, thus, could be zero—or if sampling a future position in discrete space on a lattice (where the probability of sampling **r**_*j*+1_ = **r**_*j*_ is finite) then Eq (6), could be substituted for this expression

$$f_{j}=\frac{\left(r_{j}-r_{j-1}\right)}{|r_{j}-r_{j-1}|+a}\cdot \frac{\left(h_{j}-h_{j-1}\right)}{h_{j}+1}\equiv \nabla \log h_{j}$$ (8)

However, in all of our calculations below this modification will not be needed. This is because our *h*_*j*_ has vanishingly small probability of being 0 within Δ*T* (where Δ*T* can be the camera’s frame rate in a tracking experiment) and we also sample positions in continuous space (where the probability of sampling **r**_*j* + 1_ = **r**_*j*_ is, likewise, vanishingly small).

Now, we show how all model parameters, {{*α*}, *σ*} ≡ {{*α*_0_,⋯, *α*_*m*_}, *σ*}, can be directly inferred from single cell tracking data.

#### Parameter inference from single cell tracking data

We assume the following are known from microscopy tracking data: 1) the searcher’s location (e.g. labeled bacterium) and 2), if present, the source location(s) (e.g. locations of patches of food).

To parametrize {{*α*}, *σ*}, we first write the likelihood of observing a particular bacterial trajectory

$$L\left(\left\{\alpha \right\},\sigma |{\left\{r_{i},\nabla \log h_{i}\right\}}_{i\leq j}\right)=\prod_{j}p\left(r_{j+1}|{\left\{r_{i},\nabla \log h_{i}\right\}}_{i\leq j}\right).$$ (9)

This likelihood function is parametrized in terms of the precise number of particles detected (hits) by the searcher at various points along its trajectory. While such a quantity is not directly observable, the *average* number of hits at any given location is known because the distance between the source and the searcher is known.

Thus, while we cannot maximize $L(\left\{\alpha \right\},\sigma |{\left\{r_{i},\nabla \log h_{i}\right\}}_{i\leq j})$ directly to obtain {{*α*}, *σ*} in practice, we can certainly maximize

$$\begin{array}{cc} \langle L\left(\left\{\alpha \right\},\sigma |{\left\{r_{i},\nabla \log h_{i}\right\}}_{i\leq j}\right)\rangle & ≃L(\left\{\alpha \right\},\sigma |{\left\{r_{i},\langle \nabla \log h_{i}\rangle \right\}}_{i\leq j})\\ & ≃L(\left\{\alpha \right\},\sigma |{\left\{r_{i},\nabla \log\langle h_{i}\rangle \right\}}_{i\leq j})\\ & =\prod_{j}p\left(r_{j+1}|{\left\{r_{i},\nabla \log\langle h_{i}\rangle \right\}}_{i\leq j}\right) \end{array}$$ (10)

where—in going from the first to the second equality—we have made a cumulant expansion and kept the leading order term, and going from second to third equality we used the approximation that 〈*f*(*h*_*j*_)〉 ≃ *f*(〈*h*_*j*_〉). The validity of this approximation will be assessed by first generating synthetic data where {**r**_*i*_, ∇ log *h*_*i*_}_*i*≤*j*_ are known exactly and comparing the parameters {{*α*}, *σ*} determined from the maximization of the exact likelihood function, $L(\left\{\alpha \right\},\sigma |{\left\{r_{i},\nabla \log h_{i}\right\}}_{i\leq j})$, and the approximate likelihood function, $L(\left\{\alpha \right\},\sigma |{\left\{r_{i},\nabla \log\langle h_{i}\rangle \right\}}_{i\leq j})$. As expected, we will find that the approximate likelihood function requires longer time traces before its maximum converges to the correct answer.

Now, from the fully parametrized model, we will show in the results section how we can predict the bacterium’s dynamical response to arbitrary food source configurations, food source emission rates and food source dynamics (if the food source, say, is a bacterial prey sought by a predatory bacterial searcher).

From our parametrized model, we can also infer statistical distributions that, in some circumstances, would require much more data to fully quantify than is necessary to parametrize {{*α*}, *σ*}. These include, just as examples, predictions regarding: 1) the food source ‘capture radii’ (the initial searcher-source distance at which the searcher has a 50/50 chance of finding the source in a specific number of steps); 2) both tumble angle and run length distributions in the direction of and away from a food source; and 3) the bacterium’s adaptation time (i.e. how long it takes for the bacterium to respond to ▽ log *h* or, in other words, how many initial *α*’s are zero).

For this reason, it is now convenient to introduce working definitions of *run-and-tumble* statistics that we will use in the results section. Mathematically, we define these according to a prescription provided by Berg and Brown [4].

Bacterial trajectories are random walks made of successive steps where the change in the direction is 0° to 180° from one time step to the next (time steps could be the frame rate of the camera). If multiple successive steps are straight enough, in other words, if the change in direction between multiple successive steps is small enough, they constitute a run. By definition, a run starts when the change in the direction is less than 35° for three successive steps. The end of a run is when the change in direction is more than 35° for two successive turning points, or when it was greater than 35° for one turning point and the average of the two is also greater than 35°. In addition, the tumbling angle is defined as the change in the direction from one run to the next.

#### Algorithm for generating synthetic data

**Fig 1:**
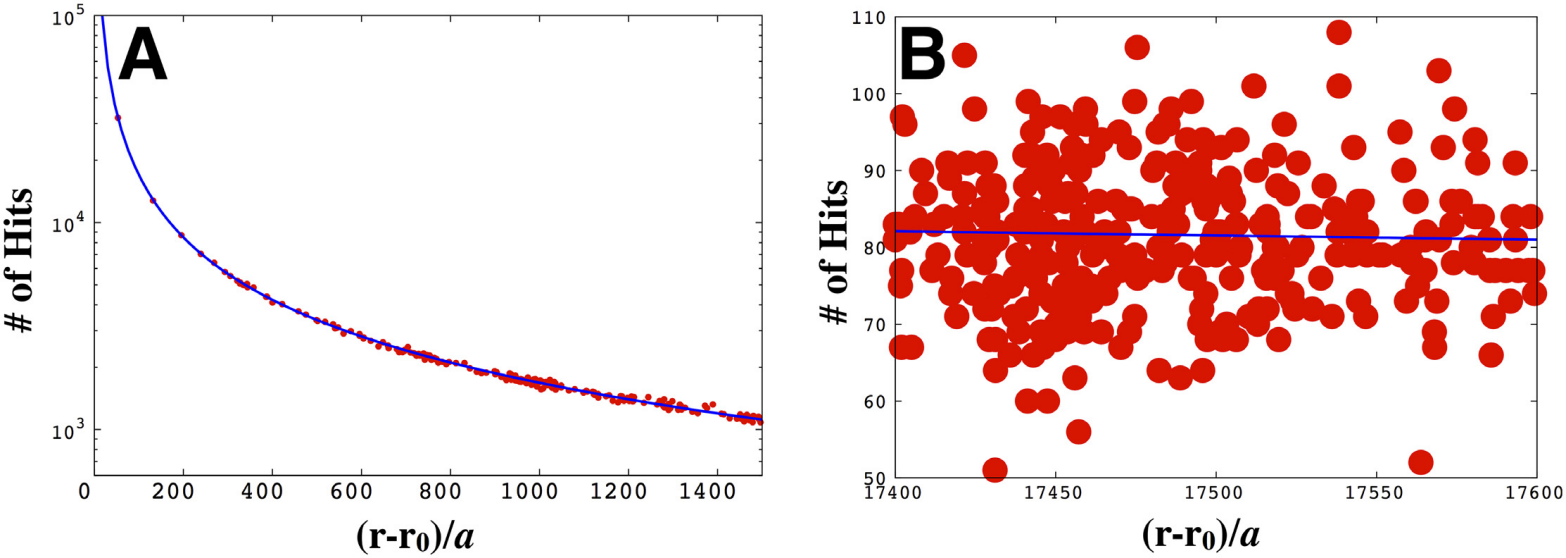
The noise in the number of hits received by bacteria increases as the distance from the source increases. The red dots in A) denote the exact number of hits the searcher collects over the course of a trajectory moving toward the point source in a log-normal plot. The blue curve is a plot of the mean number of hits expected, Δ*T* × *R*(**r**|**r**_0_), plotted against (**r** − **r**_0_)/*a*, the radial distance from the source divided by the searcher’s radius. (**r** − **r**_0_) is given in Eq (2). In B) we show a region of A) further out from the source [now on a normal plot] exhibiting high fluctuations in the number of hits. We used Δ*T* = 0.1*s*, *a* = 1*μm*, $R=1.7\times 10^{7}s^{-1}$, and *λ* = 10^5^ *μm*.

To benchmark our method, we generated stochastic bacterial trajectories—that serve as a proxy for single cell tracking data—following these steps:

1. we compute the searcher’s mean hits received at its current position **r**_*j*_ over some interval Δ*T*, $\bar{h_{j}}=\int_{t}^{t+\Delta T}dt^{\prime }R\left(r_{j}|r_{s};t^{\prime }\right)$, where Δ*T* is an integration time (for example, it can be on the order of 0.1*s* which is a typical tumbling time [11]).
2. we sample a stochastic hit value, *h*_*j*_, received at the position of the searcher **r**_*j*_, from the Poisson distribution ($P\left(h_{j}\right)=\frac{e^{\left(-{\bar{h}}_{j}\right)}{\left(\bar{h_{j}}\right)}^{h_{j}}}{(h_{j})!}$). As an illustration, the number of stochastic hits plotted versus radial distance from a point source is shown in Fig 1;
3. using this hit value (as well as previous hit values and previous positions), we sample the position of the next step, **r**_*j* + 1_, from the transition probability given in Eq (5); and
4. repeat the previous steps until the searcher reaches the source or, alternatively, a predefined distance from the source.

Given this synthetic trace, we maximize the likelihood (or, technically, the log likelihood) with respect to the model parameters {{*α*}, *σ*} via a standard grid search [by scanning over all possible values of the parameters and picking those values that maximize the likelihood]. We’ve also maximized our likelihood function using simple Monte Carlo though the real advantage of this approximate method is realized in cases where we assume a large number of parameters (i.e. if we have a long memory with many *α*’s).

## Results

Our results are broken down along the following topics:

1. Role of memory on bacterial behavior, Fig 2;
2. Model parameter inference from synthetic single cell tracking data, Figs 3–6;
3. Predicting bacterial behavior in different source configurations, Figs 7–11.

**Fig 2:**
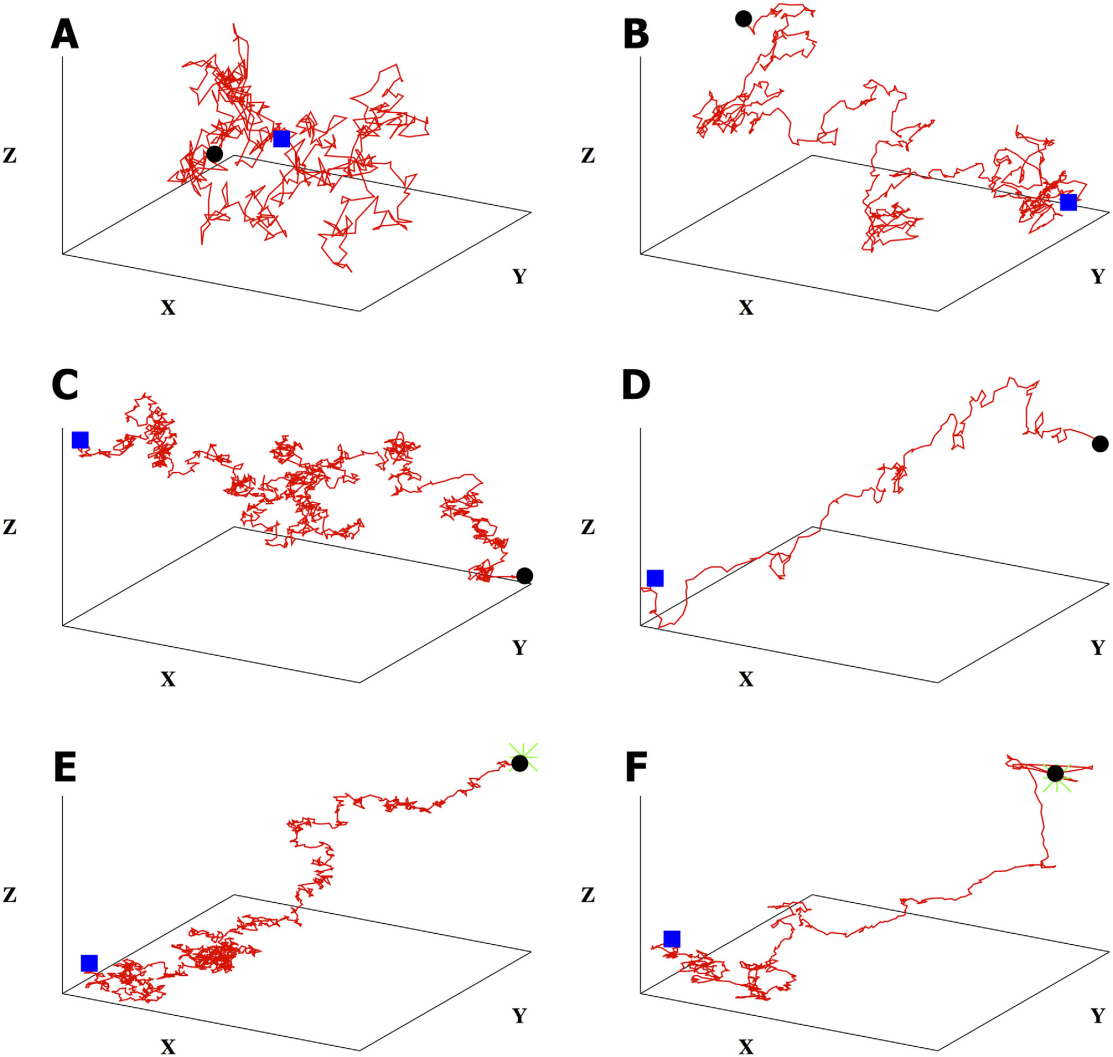
Memory of previous hits introduces qualitative differences in bacterial trajectories. All trajectories start from the blue square and end with the black circle. We have defined *X* *≡* *x/a, Y* *≡* *y/a,* and *Z* *≡* *z/a.* The green star in the last two plots denotes the point source’s location. Here we show typical bacterial trajectories generated from our model (Eq (5)) with: A) no gradient and one-step memory; B) no gradient and a memory of *m* = 5; C) a linear gradient (*x* direction) and one-step memory; D) a linear gradient (*x* direction) and memory of *m* = 5; E) the presence of a food gradient due to a point source at (1000, 1000, 1000)*a* and one-step memory. The searcher starts at (0, 0, 0)*a* and locates the source; F) same as in e) except that we have a memory of *m* = 5. When there is a source, we stop all trajectories when the distance between the searcher and the source is less than 60*a*. We used *α*_0_/*a* = 130 and *σ*/*a* = 10 throughout.

### 1. Role of memory on bacterial behavior

Before we discuss parameter inference, we briefly highlight qualitative new trends in Fig 2 that arise in the presence of memory, *m* as defined in Eq (5), that do not explicitly depend on the memory’s precise numerical value. For this reason, in this subsection we only consider bacterial trajectories where the parameters *α*_*i*_ are independent of the index *i* and are positive (implying the presence of a CA as opposed to a CR).

Fig 2A and 2B explore the effect of memory from which, as we will show later, emerge *run-and-tumble* statistics. In particular, Fig 2A shows that in the absence of a food gradient with one-step memory—the case where m = 0 from Eq (5)—the trajectory is, predictably, a random walk with no preferred direction. However, with memory and no gradient as in Fig 2B, the searcher shows increased run lengths.

Fig 2C and 2D highlight the searcher’s behavior in the presence of a *linear gradient* with and without memory. As expected, the searcher now exhibits a directional bias (in the direction of increasing food concentration) however, as we will discuss later, tumble angles and run lengths are stationary in time with a linear gradient. In the presence of *linear gradient*, the searcher shows decreased tumbling angles and increased run lengths with memory.

Tumble angles and run lengths are no longer stationary along the trajectory in the presence of a point food source denoted by the green star in Fig 2E and 2F. We will demonstrate this quantitatively later in Fig 9. Also, the bacterium locates the source exclusively through stochastic CA detection. The probability of locating the source depends on parameter values (which we later explore in Fig 10).

### 2. Model parameter inference

**Fig 3:**
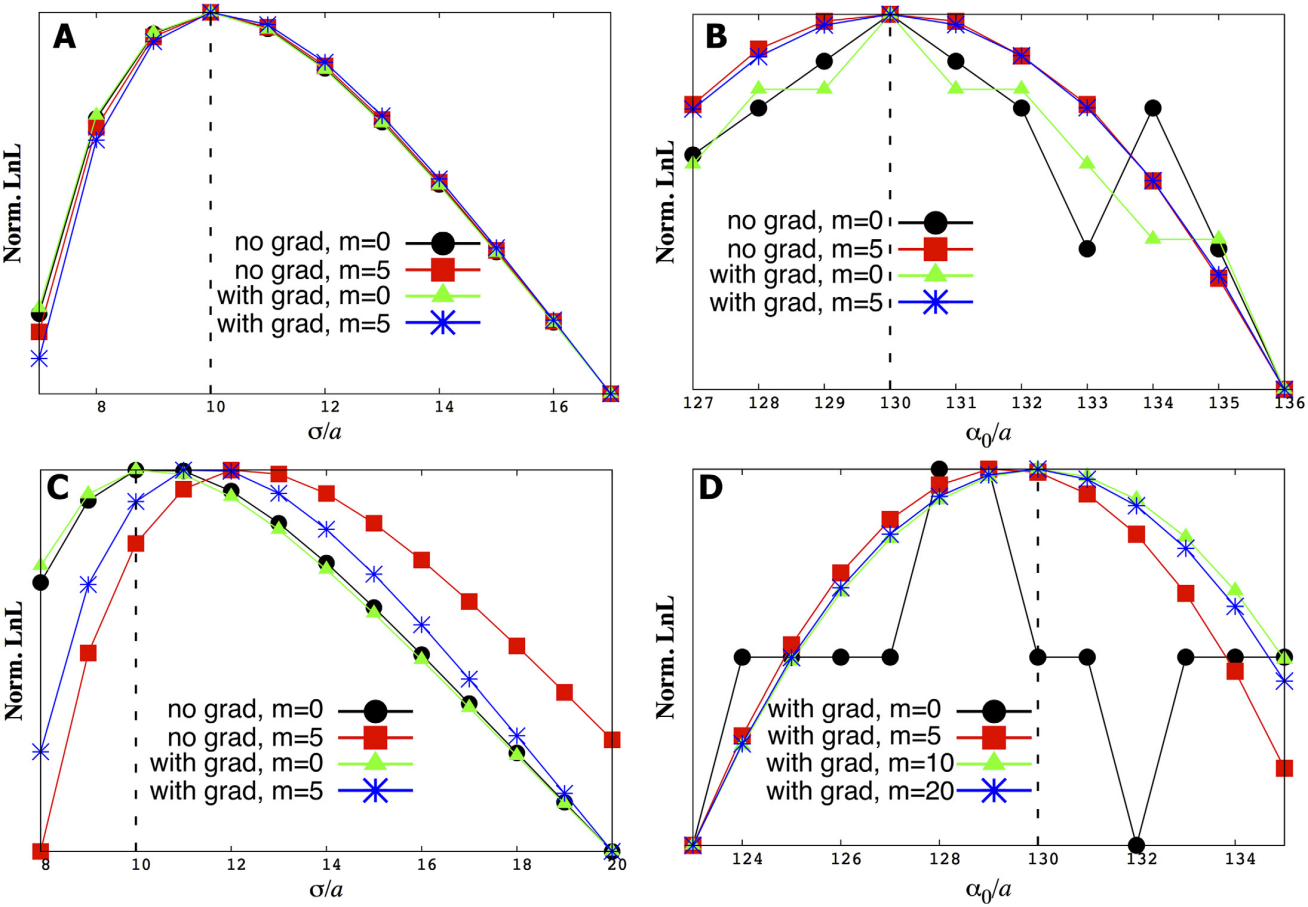
We maximize the likelihood function, Eq (9), to find both *σ* and *α*_0_. As an illustration, we show slices of the likelihood function where one variable is held fixed. For instance, in A) and C) *α*_0_ held fixed while in B) and D) *σ* held fixed. In general, we do a full two-dimensional scan to determine both *σ* and *α*_0_ simultaneously. Our estimates coincide with the correct theoretical value used to generate the original synthetic trajectory (vertical dashed line at *σ*/*a* = 10 and *α*_0_/*a* = 130). We tested our method under a variety of conditions. In particular, ‘with grad’ means in the presence of a point source. A) and B) are inferences made using the exact number of hits while C) and D) are made using the average number of hits.

**Fig 4:**
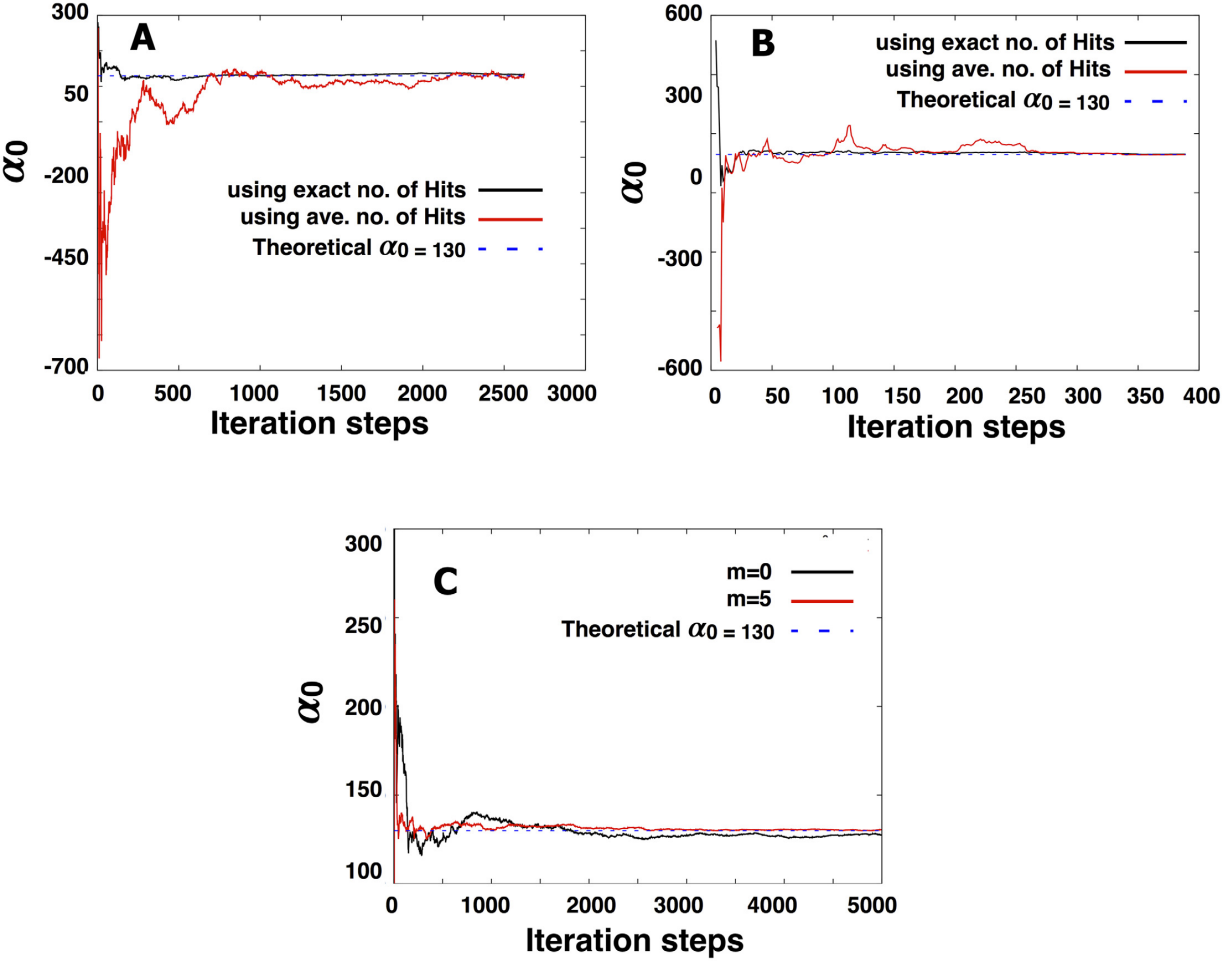
Within a few hundred iteration steps, our parameter estimates converge to the correct theoretical value whether we use the exact (Eq (9)) or approximate (Eq (10)) likelihood function. Iteration steps are the Δ*T*’s along the trajectories used to make the point estimate of our parameters. In A) and B) we consider a point source without memory (*m* = 0) and with memory (*m* = 5), respectively. The results of these calculations confirm that our first cumulant approximation of the likelihood function (shown in Eq (10)) eventually converges to the correct theoretical parameter values. In C) we show the same results in the absence of a gradient using the exact number of hits.

In Fig 3 we show the estimates of the model parameters extracted from trajectories such as those shown in Fig 2. The dotted lines (theoretical values used to generate the data) are in excellent agreement with the values inferred from the synthetic data. This agreement, tested for different parameter values, validates our first cumulant approximation, as detailed by Eq (10). In addition, Fig 4 shows the time (or trajectory length) needed for the results to converge.

**Fig 5:**
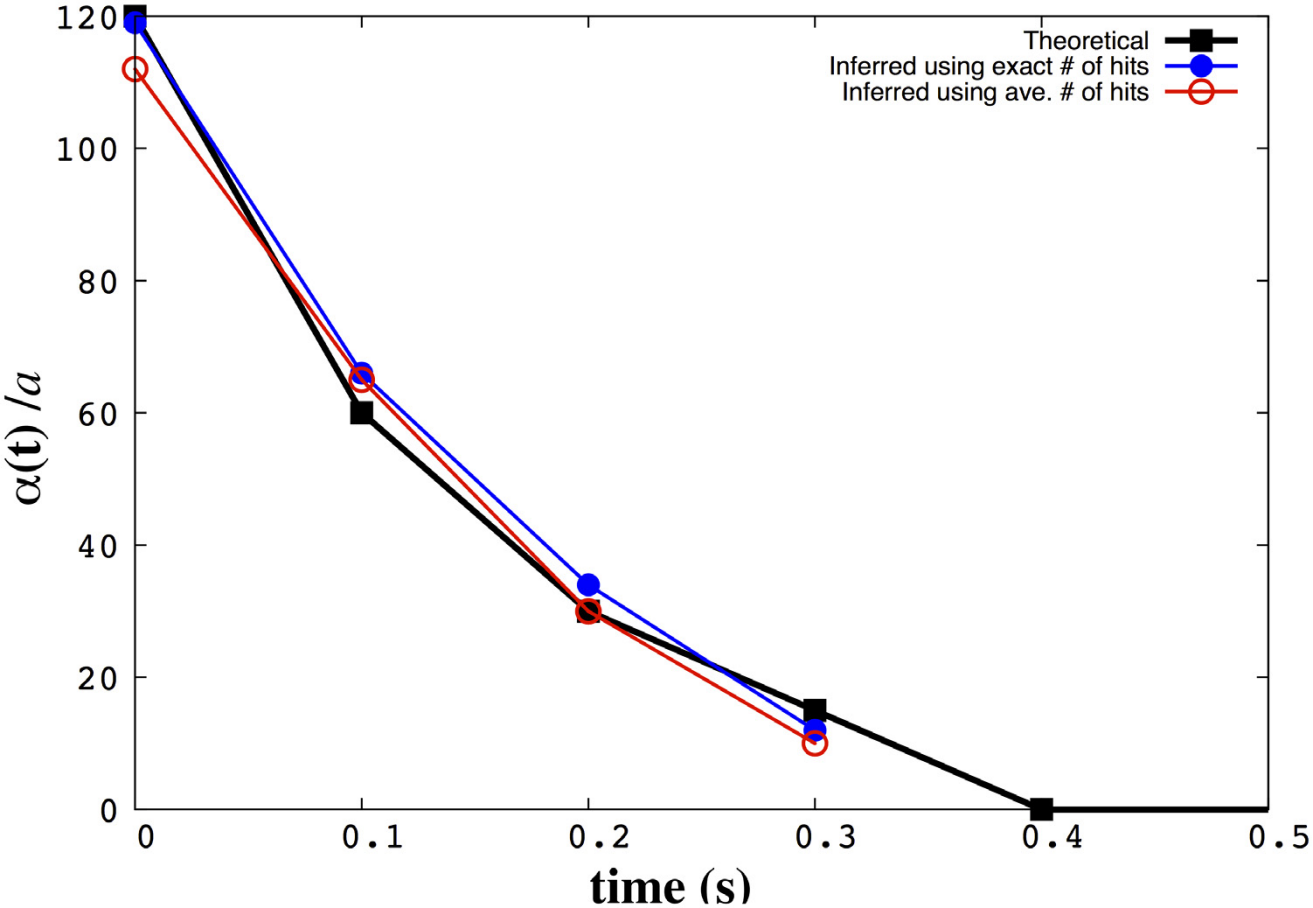
We can infer model parameters for non-constant memory. We consider the case of decaying memory ($\alpha_{i}=\frac{1}{2^{i}}\alpha_{0}$) and infer (*α*_0_, *α*_1_, *α*_2_, *α*_3_) using both exact and average number of hits as indicated in the figure’s inset, and compare our estimates to their correct theoretical values used to generate the synthetic data. As the number of parameters we need to estimate from the data increases, we need longer trajectories to obtain accurate estimates.

Furthermore, we considered the case of non-uniform memory. First, we chose to make the bacterium’s memory decay monotonically (that is, *α*_*i*_ = *α*_0_/2^*i*^), and we inferred model parameters, (*α*_0_, *α*_1_, *α*_2_, *α*_3_), for the case of *m* = 3 as shown in Fig 5.

**Fig 6:**
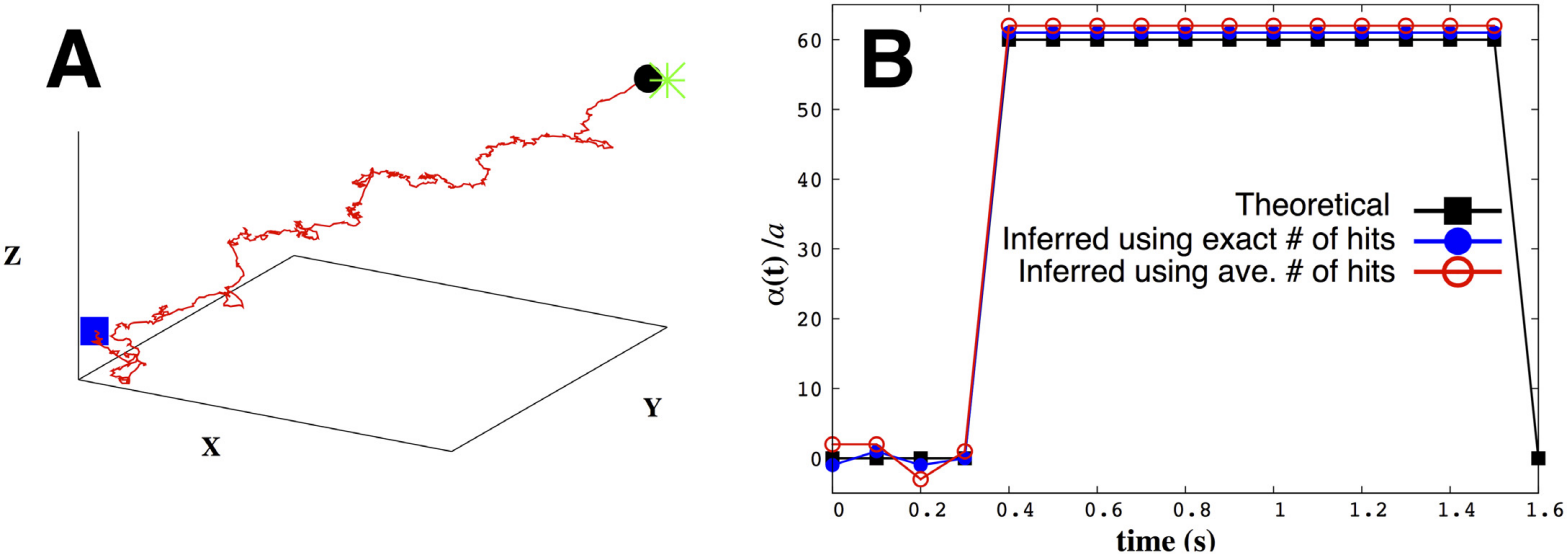
We can infer adaptation times. We consider a theoretical adaptation time of 3Δ*T* (by setting *α*_0_ = *α*_1_ = *α*_2_ = *α*_3_ = 0 and *α*_4_ = *α*_5_ = … = *α*_15_ = *const* = 60*a*). In A) we show a typical trajectory given these {*α*} values. In B) we show both theoretical {*α*}’s (as black squares), our inferred values using the exact hits (as blue dots) as well as using the average number of hits (as red circles). The blue square (in A) shows the start of the trajectory at the origin, the black dot shows its end, and the green star denotes the point source’s location at (1000, 1000, 1000)*a*. We used *σ*/*a* = 5 and stopped the trajectory when the searcher was at a distance 50*a* from the point source. We inferred *α*_0_ through *α*_3_ individually (as would be necessary in estimating adaptation times from single cell tracking data) but assumed *α*_4_ = *α*_5_ = … = *α*_15_ = *const* and inferred them as a single parameter.

Second, we considered the case where the first few *α*_*i*_’s are zero. This represents the physically relevant effect of a finite adaptation time [29, 33]. That is, the case where the bacterium responds to the gradient at some point in the past though not the immediate past. Thus, there is a delay in the bacterium’s response to ▽ log *h*. Fig 6 is an important result of our paper. It shows that we can successfully estimate the bacterium’s adaptation time (i.e. estimate the bacterium’s delay in response to the local gradient).

### 3. Predicting bacterial behavior

**Fig 7:**
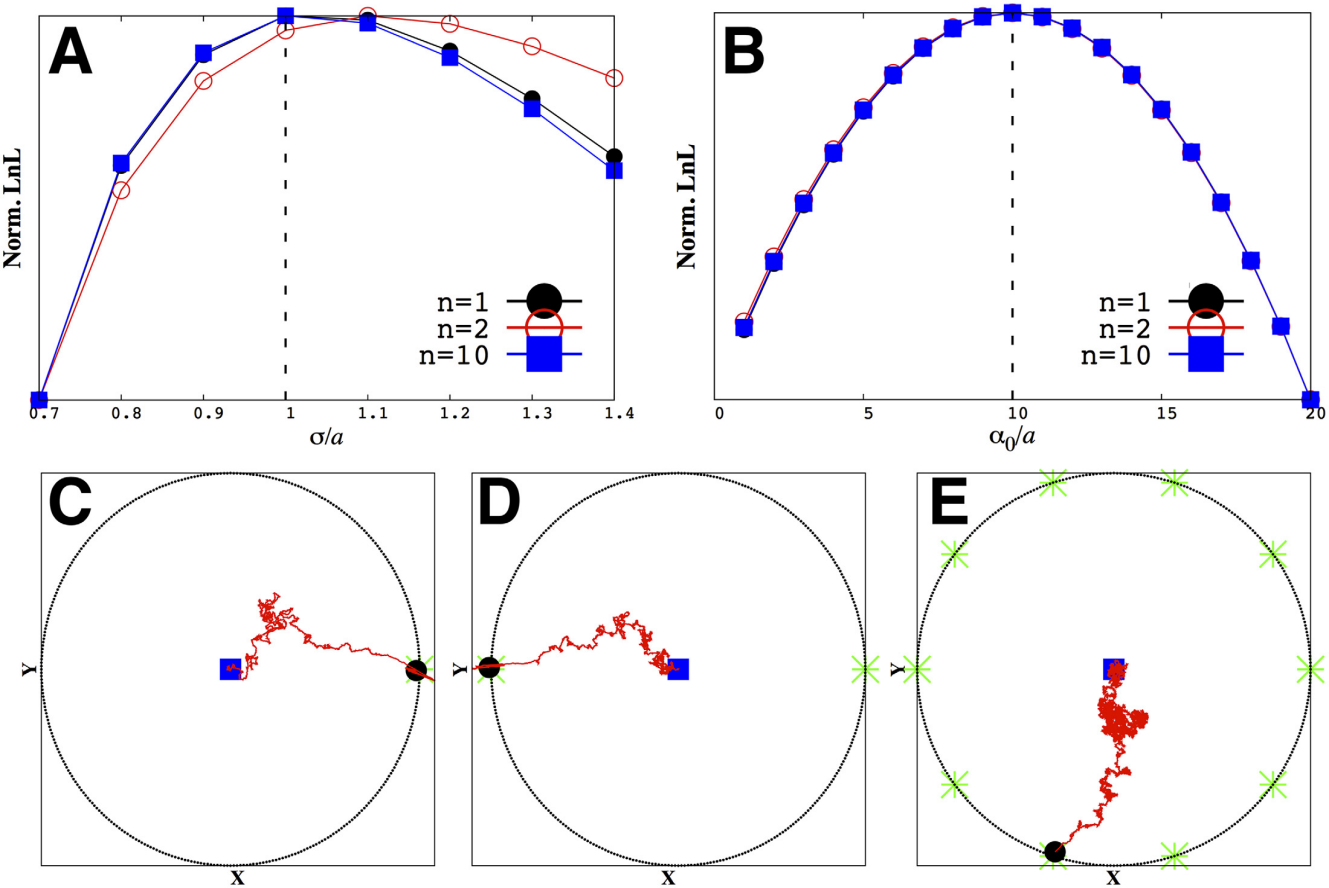
Our model parameters are insensitive to the particularities of the source configuration. In A and B we show our model parameter inference using the average number of hits for different CA profiles (*n* = 1, 2 and 10 sources). We infer the same parameters no matter the configuration of the sources around the searcher. Typical trajectories are shown in C) through E). As before, the vertical dashed lines in A and B show the correct theoretical parameters values (*σ*/*a* = 1, *α*_0_/*a* = 10). We used a memory of *m* = 30 and stopped the trajectory when the searcher was at a distance 5*a* from the point source. Again the blue square shows the start of any trajectory at the origin, the black dot shows its end, and the green star(s) denotes the point source’s location. The radius, *R*, of the circle on which the point sources -symmetrically- lie in C) through E) is 1000*a*. The searcher always starts from the center of this circle shown by the blue square.

Fig 7 also captures a central result of our paper. We show that the model parameters ({*α*}, *σ*) we extract are independent of the source configuration even in the presence of large external noise and non-uniform gradients. This outcome is critical in proving that models parametrized in one source configuration can be used to make predictions about other (perhaps more interesting but less well controlled) source configurations.

Thus, concretely, the information we gather on the model parameters from a single source around the bacterium would be sufficient to predict how the bacterium would behave around two and even ten sources; see Fig 7 for details.

We emphasize that by contrast to other inference methods for chemotaxis parameters that rely on well-controlled gradients [25], our results hold even if, as is the case of a bacterial predator, the bacterium is only attracted to point sources (such as prey) where gradients are not well-defined. What is more, we do not impose *run-and-tumble* dynamics *a priori*.

**Fig 8:**
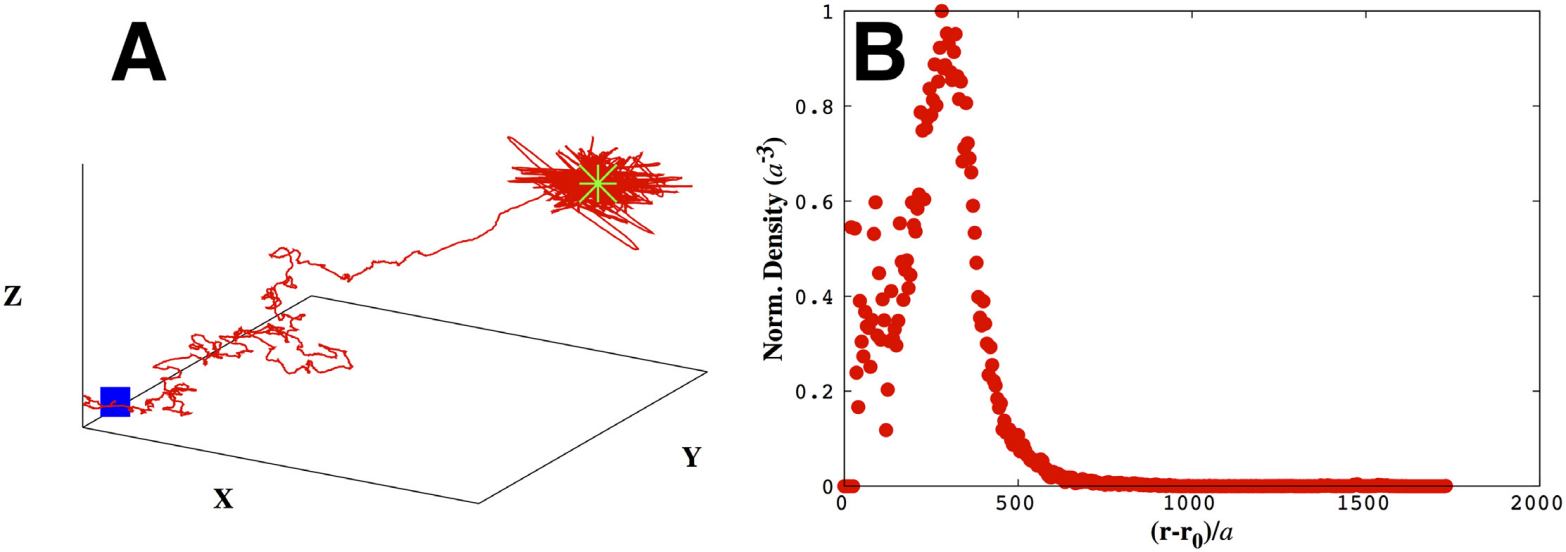
The volcano effect emerges from our model as a consequence of the rapid CA concentration variation near the source. In A) we show a typical trajectory for a bacterium (starting at the origin) exhibiting erratic behavior near the point source ((1000, 1000, 1000)*a* denoted by the green star). In B) we show the probability of finding the searcher as a function of radial distance from the source. The resulting density profile—resembling the mouth of a volcano in 2d [36, 37]—has maximal bacterial densities occurring on a ring around the point source. Here we used *m* = 30, *α*_0_/*a* = 30, *σ*/*a* = 1 and *X* ≡ *x*/*a*, *Y* ≡ *y*/*a*, and *Z* ≡ *z*/*a* and stopped the trajectory at 12000 steps. We’ve normalized the value for the density at *r*−*r*_0_ by the volume enclosed in the shell at that distance to ensure that our results are independent of volume.

**Fig 9:**
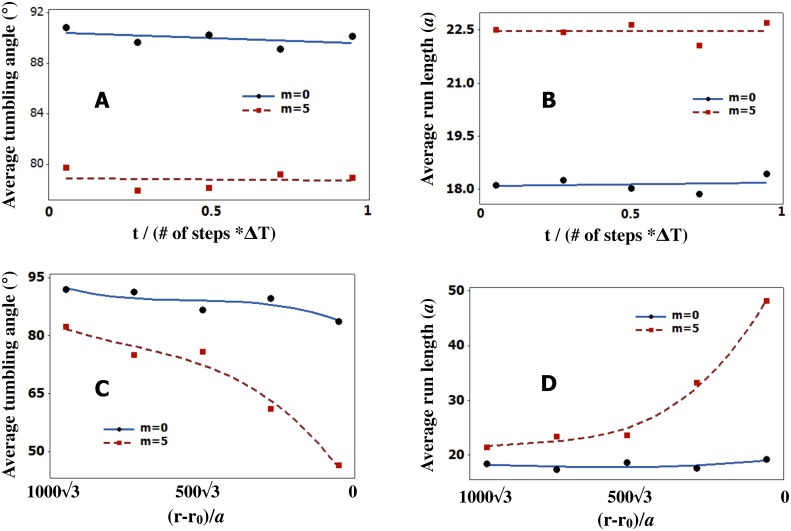
*Run-and-tumble* statistics are not stationary in the presence of a point food source. They depend on the bacterium’s distance from the source. The average tumbling angle, in A), and the average run length, in B), remain roughly constant in time in the absence of gradient. We show plots for one step memory (*m* = 0) as well as *m* = 5. However, when a point source is introduced, the average tumbling angle, in C), as well as the average run length, in D), change as a function of distance from the point source as the searcher moves from the starting point toward the source. For all plots we used *α*_0_/*a* = 130 and *σ*/*a* = 10 as well as Δ*T* = 0.1*s*.

**Fig 10:**
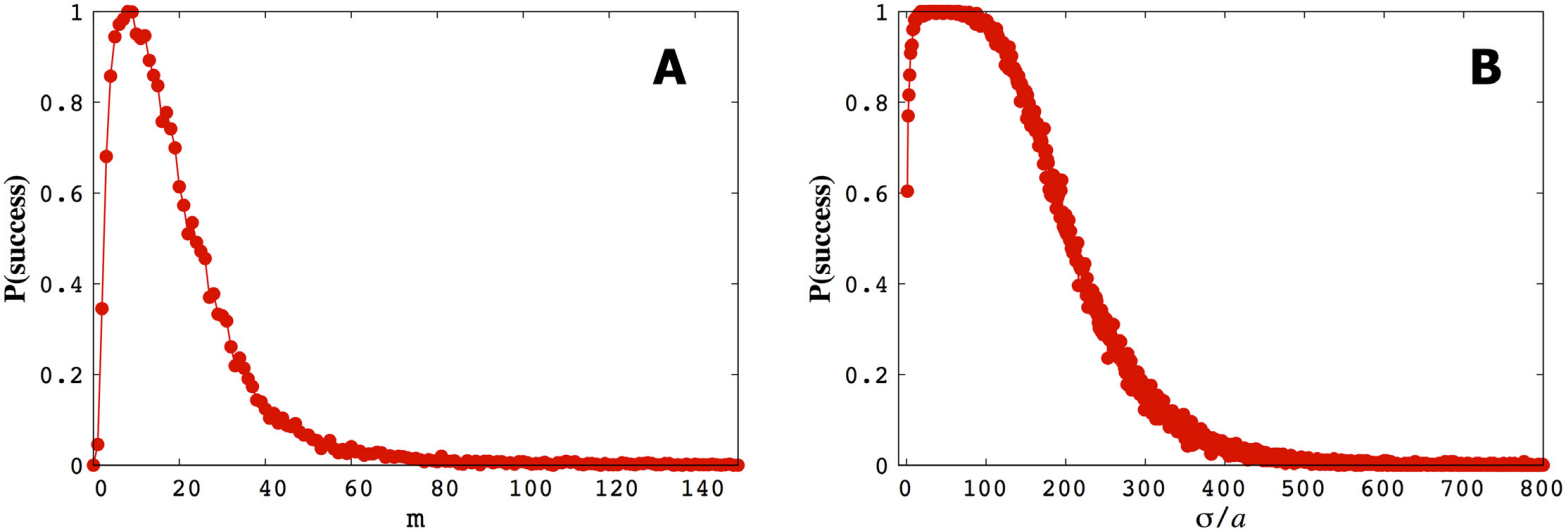
The probability of locating a point source is a non-monotonic function of both memory and precision. In A) we show how the probability of successfully locating a source varies with memory, *m* (using *σ*/*a* = 10 and *α*_0_/*a* = 130). The searcher starts at (0, 0, 0)*a* and the point source is located at (10000, 10000, 10000)*a*. We call a trajectory successful if, within 50000Δ*T* the searcher gets to within 30*a* of the point source. In B) we show the probability of success versus *σ* under the same conditions as in A). We discuss why these distributions are non-monotonic in the main body.

There are a number of statistical signatures of a targeted search by a bacterium that we can now quantitate that depend on features such as, for instance, the length scale over which a source’s gradient varies dramatically rather than the particularities of the signaling pathway responsible for chemotaxis. Here are four such signatures:

1. In the immediate vicinity of the source, the searcher’s trajectory becomes erratic, see Fig 8. That is, bacteria overshoot the source and turn back. This arises because the CA gradient varies very rapidly with distance in that neighborhood. For instance, the rate function (Eq (2)) increases by as much as 10% for a small displacement by the searcher of just one body length when it is about ten body lengths away from the source (see, for example, Fig 1A). Interestingly, in 1901, in a capillary tube experiment reminiscent of a point food source, it was shown that bacteria swam past high concentration regions neighboring the capillary before turning back [34, 35].
2. The “volcano effect”, describing how bacteria cluster near but not on a point source [36, 37], emerges from our model. As a consequence of the bacterium overshooting the source and re-directing its search, the bacterium spends most of its time on the approximate surface of a sphere surrounding the source, see Fig 8.
3. *Run-and-tumble* statistics are not stationary as the searcher approaches the point source, see Fig 9. In other words runs, on average, get longer and tumbling angles, on average, get smaller in a predictable way. The further away the searcher is from the source, the fewer hits it receives, the more tumbles it takes per unit time interval. The change in tumble and run statistics can, just like the volcano effect, be indicative of a targeted search by the bacterium.
4. Given too large a memory (or too little a *σ*), the bacterium initially overcommits to a particular direction and requires a prohibitive amount of information to re-direct its search. Predictably, given too little memory (or, equivalently, too large a *σ*) a bacterium searches randomly. Thus the probability of finding a point source is a non-monotonic function of memory and precision, see Fig 10.

**Fig 11:**
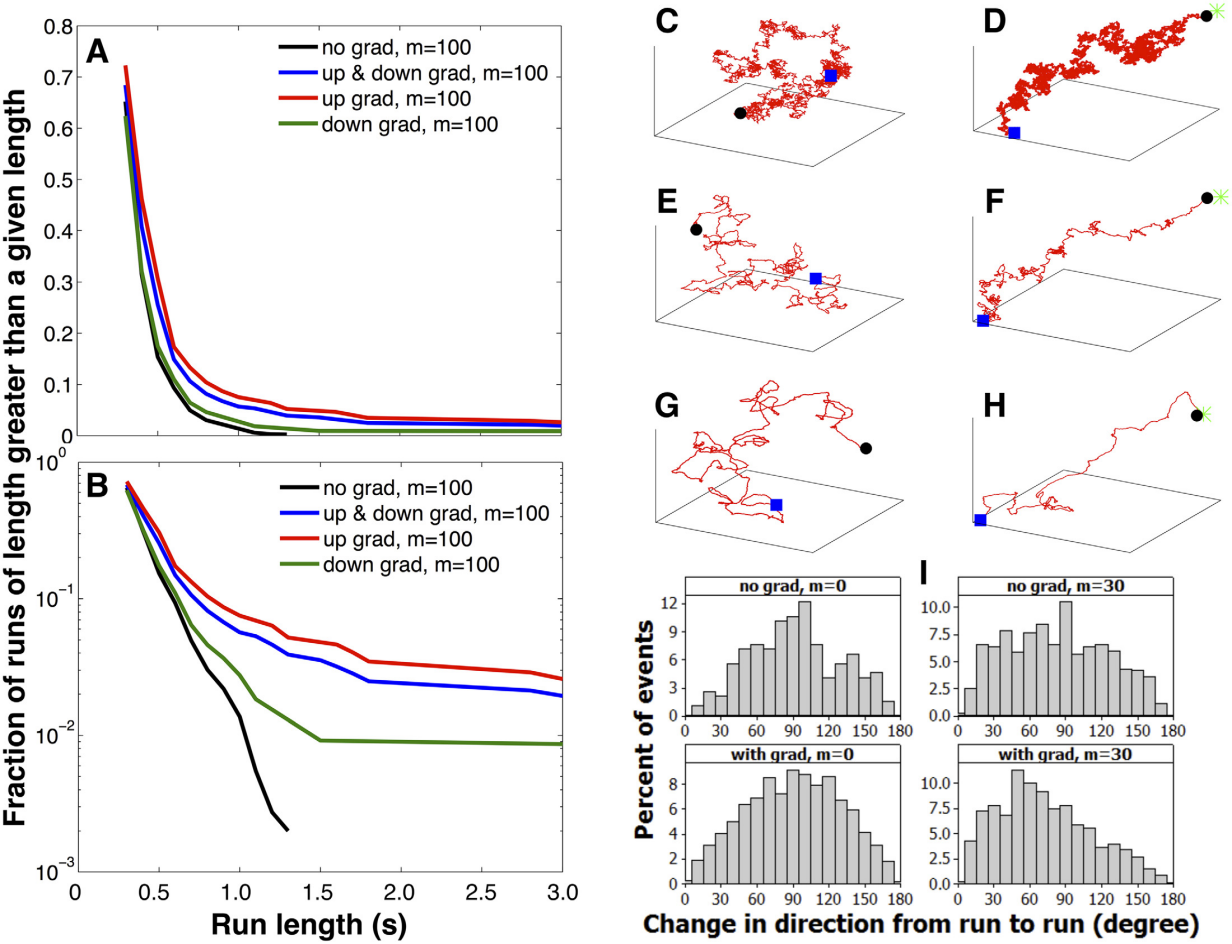
Run-and-tumble behavior emerges from our model. Here we illustrate how *run-and-tumble* statistics emerge from our model by focusing on representative statistics of our trajectories. Comparison between the plots described below and experiments are discussed in the main body. In A) and B) we show run durations (run lengths in units of seconds) distributions in linear and log scale, respectively, under various conditions (in the absence and presence of gradient for a memory of *m* = 100). By construction, the shortest runs are 0.3*s* long (as per the definition of a run in the main body). In C), E), and G) we show the corresponding trajectories in the absence of gradient for the case of one-step memory (*m* = 0), when *m* = 30, and when *m* = 100, respectively. In D), F), and H) we show the corresponding trajectories in the presence of gradient due to a point source again for *m* = 0, *m* = 30, and *m* = 100, respectively. In I) we histogram changes in direction between the end of one run and the beginning of the next in the absence and presence of gradient with and without memory. For all trajectories, we used *α*_0_/*a* = 10 and *σ*/*a* = 1. The point source location is at (1000, 1000, 1000)*a*. Again, the start of all trajectories is shown as a blue square, its end as a black circle. The green star denotes the location of the point source. Furthermore, ‘no grad’ indicates that there is no food source and ‘with grad’ indicates the presence of a point source. Details are discussed in the main body.

We highlight that *run-and-tumble* behavior is not imposed on our model by hand. Rather this behavior qualitatively emerges as a consequence of memory and either stochasticity of the input or precision with which the bacterium integrates the input and converts hits into a directional bias. In particular we briefly compare our results to the well-established observations in the original *run-and-tumble* literature:

1. Berg and Brown [4] found that for wild type *E. coli*, the distribution of tumble lengths as well as the distribution of run lengths is approximately exponential, the shortest tumbles and the shortest runs being the most probable. Tu and coworkers [2] also found similar run length distributions (Figs 4–6 in Ref. [4] and Fig 4B in Ref. [2]). These observations are recapitulated in our Fig 11A and 11B which show that, under a broad set of parameter values, the same behavior is also observed from our model. Just as in the real data (Figs 6 in Ref. [4]) our run distributions are not perfectly linear on a log-normal plot. That is, they are not perfect exponentials.
2. Tu and coworkers [2] found that overall average run length in an exponential gradient is longer than that in a homogeneous environment. This is also reproduced in our model as shown in Fig 11A and 11B because runs down a gradient are comparable to runs in homogenous environments while runs up a gradient are typically longer. That is, the overall average run length is increased in the presence of gradients. This is also consistent with Berg and Brown’s results in Ref. [4] where they observed that up gradient run lengths (i.e. runs that move up the gradient) are typically longer than the down gradient run lengths while the down gradient run length distribution is similar to that of the run length distribution in the absence of gradient. For example, see Fig 6 (bottom) in Ref. [4], and Fig 4B in Ref. [2]). In experiments for some CAs, runs down gradients can be longer than runs without gradient (Fig 6 (bottom) in Ref. [4]). Nonetheless it still holds that such down gradient runs are still typically smaller than runs up gradient. Our model is consistent with these overall observations (Fig 11A and 11B).
3. Berg and Brown [4] as well as Buguin and coworkers [11] studied (experimentally and theoretically, respectively) the distribution of bacterial reorientation during tumbling. For instance, Berg and Brown observed a mean angle change from run to run significantly below 90° in the presence of a gradient (62° ± 26° in Fig 3 in Ref. [4]). Our tumbling angle distributions in Fig 11 are broadly consistent with these observations [4] and the breadth of our distribution is sensitive to the precise numerical value assigned to our memory parameters. By contrast, in the absence of a CA/CR gradients, reorientations of the bacterium are random, resulting in a mean angular change of about 90° between successive steps (runs) and this is also consistent with observation [4].
4. Finally, by construction, our model is consistent with the observation that effects from various sources of CA or CR are additive [16].

## Discussion

Berg and Brown’s original single particle tracking analysis of *E. coli* [4, 16] not only shed light on *E. coli*’s *run-and-tumble* dynamics but also directly motivated the types of models proposed in subsequent decades [2, 37–42]. Since then, the signaling pathway responsible for *E. coli*’s chemotactic response has been extensively studied [2, 30, 37, 38, 43] and attention has been focused on internal noise sources arising from the stochasticity of the signaling pathway [22, 44, 45].

While the biochemical reactions responsible for chemotaxis in some bacteria are well understood [4, 24, 38] the chemotactic behavior of others—such as that of the model bacterial predator *Bdellovibrio bacteriovorus* that preys upon *E. coli*—remain elusive [46, 47].

Here our strategy is to extend the theoretical body of work—and inference work in chemotaxis in particular [25]—to study the regime where external (detection) noise is treated explicitly. Since we would like our theory to be valid for bacterial species whose chemotactic signaling network is not well characterized, we do not treat internal noise sources explicitly. Instead, internal noise is treated implicitly through the phenomenological precision parameter *σ* which we directly infer from the data. In other words, the precision parameter implicitly accounts for the noise along the steps of the complex reaction network responsible for signal transduction from the chemoreceptors to the bacterium’s flagella [8, 32, 40, 48] as well as the noise due to Brownian motion of the bacterium in its environment. As a result, our ‘top-down’ approach should be broadly applicable across bacterial species but cannot make molecular-level predictions.

As input to our model, we have used the fact that bacteria show adaptation [24, 29, 30, 38], employ a temporal sensing mechanism and have a memory of previous events [3]. We do not assume two-state *run-and-tumble* dynamics *a priori* either [25].

Mathematically, our model captures the bacterium’s dynamics using a transition probability, Eq (5), which selects the bacterium’s preferred direction within some precision, *σ*, given memory coefficients, {*α*_*i*_}, which are all to be determined using an *inverse* (maximum likelihood) approach from single cell tracking data. Thus, we avoid indeterminable and unobservable adjustable parameters that often appear in ‘forward’ modeling methods [38]. That is, models whose form or parameters are not explicitly inferred from data.

*Run-and-tumble* statistics (including whole distributions over trajectories up and down concentration gradients) then qualitatively arise from our model from basic, physically motivated, principles of chemotaxis. What is more, our model captures—at the whole cell rather than at the biochemical level—critical features that help establish statistical signatures of targeted search by bacteria towards point sources (such as motion toward bacterial prey by predatory bacteria if predatory bacteria are attracted to CAs released by the prey). That is, our model makes explicit predictions about the dynamical behavior of bacteria even if external noise is high. For instance, the volcano effect emerges from our model as a consequence of the distance over which the gradient varies neighboring a point source. In addition, our model shows that if bacteria are tracking point sources then they should show changes in run and tumble statistics as they approach the source that we can theoretically anticipate from the normal diffusive behavior of the CAs.

More interestingly, our model provides a framework to investigate any arbitrarily complex CA/CR arrangement once our model is parametrized. Thus, it is convenient to parametrize a model in simple (presumably well-controlled) environments to then make predictions about more complex environments. In addition, and equally importantly, we can infer adaptation times even in the high external noise limit.

For the moment, our model does not treat source-searcher interaction. However, it is conceivable that a bacterial prey may detect a bacterial predator and respond. Our model is, in principle, generalizable to dynamical food sources as well as interacting sources and searchers. This direction will be the focus of future work.
